# The Application of China’s Proactive Health Management Model for Community-Dwelling Elderly in Mental Health

**DOI:** 10.31083/AP38787

**Published:** 2025-02-28

**Authors:** Huang Lin, Shujuan Xiao, Jinguo Zhai, Chichen Zhang

**Affiliations:** ^1^School of Nursing, Southern Medical University, 510515 Guangzhou, Guangdong, China; ^2^Key Laboratory of Philosophy and Social Sciences of Guangdong Higher Education Institutions for Collaborative Innovation of Health Management Policy and Precision Health Services, 510515 Guangzhou, Guangdong, China; ^3^School of Health Management, Southern Medical University, 510515 Guangzhou, Guangdong, China

As population aging becomes a global challenge, China, with the largest and fastest aging population, is facing an unprecedented health crisis. In China, an estimated 173 million adults grappling with mental disorders, with a concerning 158 million among them never having sought or received any form of professional assistance for their conditions [1]. A study shows that 15.07 million people aged 60 or older in China have suffered from dementia, 9.83 million suffered from Alzheimer’s disease, 3.92 million suffered from vascular dementia, and 1.32 million suffer from other forms of dementia. The overall prevalence of mild cognitive impairment is estimated to be 38.77 million [2].

Mental disorders have become more common in China. Rapid social change is a probable cause of the general increase in psychological stress and tension. Mental disorders impose a substantial disease burden on the Chinese government [3]. Addressing the mental health of the elderly in China is urgent due to practical concerns. The mental health protection of the elderly is not only related to the lives of the elderly population, but also to the steady and healthy development of China’s elderly care industry, which is a major issue concerning the national economy and people’s livelihood. As the elderly population grows, the contradiction between their increasing demand for mental health services and the limited supply of social support resources will become increasingly prominent. Communities, as the main place for the elderly, shoulder the significant responsibility for the elderly health issues. Therefore, it is imperative to invest in mental health research and develop a proactive health management model tailored specifically for the community-dwelling elderly in China, one that not only addresses the health needs of the people but also aligns with the country’s national strategic development. China has the capacity to enhance not merely the well-being of its own vast populace, but also the lives of individuals globally who struggle with mental health conditions [4].

The Comprehensive Mental Health Action Plan 2013–2030 had two of four objectives focused on providing comprehensive, integrated, and responsive mental health and social care services in community-based settings and strengthening information systems, evidence, and research for mental health. The World Health Organization recommends developing comprehensive community-based mental health and social care services and the promotion of self-care, engaging service users and family members and/or carers with practical experience as peer support workers, using evidence-based innovative approaches to provide psychological support at scale, and building up interdisciplinary community-based mental health services for people across the life-course, through for instance outreach services, home care and support, primary health care, emergency care and community-based rehabilitation [5]. In recent years, the Chinese government has prioritized these two objectives in enhancing the mental health service system and issued a series of policy projects.

The proactive health management model for community-dwelling elderly (CDE-PHM model), established in Guangdong, China, also meets the requirements of key goals to a certain extent and has achieved certain results in improving the mental health among elderly people in the community. The empirical research of the CDE-PHM model was based on the Community Elderly Health and Behavior Panel Study (CEHBPS) project, an interdisciplinary longitudinal survey project conducted by the Health Management Strategy and Policy Research Innovation Team (CHealth-HMSP) of Southern Medical University. At the time of the article’s publication, a total of 3392 elderly were included, and their baseline data was used to construct a proactive healthy index for community-dwelling elderly (PHICE), analyzing the characteristics and levels of proactive health management among the elderly. Based on these characteristics and index results, the implementation path of proactive health management for the elderly was drawn up by the panel discussion, and a specific intervention scheme was determined. Two communities were randomly selected from the CEHBPS cohort as the intervention group and the control group. The differences between the two groups were analyzed and the practical effectiveness of the model was evaluated by using propensity score matching and differences-in-differences (PSM-DID) after six months of model intervention. After the six month implementation of the CDE-PHM model, the self-assessed health status, social network level and self-efficacy of the elderly in the intervention group were significantly improved, and their mental health disorders such as depression and psychological distress were alleviated. The CDE-PHM model advocated fostering elderly individuals’ active engagement in social activities, enhancing emotional support networks among family and friends, improving self-efficacy and cultivating positive psychology. It also promoted maintaining a balanced and scientific diet (e.g., increasing the daily consumption of fresh fruits and vegetables), ensuring adequate sleep of 7 to 8 hours per day, and fostering health awareness while encouraging the habit of timely medical treatment for physical discomfort. These comprehensive measures, working in synergy, significantly elevate their self-efficacy and overall happiness, effectively alleviating mental health disorders and ultimately promoting a proactive and resilient approach to health among the elderly population. These results indicate that the CDE-PHM model has shown initial effectiveness and has promoted the improvement of the provision and quality of community-based services for mental health. The CDE-PHM model is currently being continuously promoted for its application and development.

In the past few years, China has undergone profound social transformations, steadily fostering a greater appreciation for the significance of mental health. At the same time, the country’s remarkable economic advancements have paved the way for allocating additional resources towards enhancing mental health services. Nevertheless, transforming this heightened awareness into tangible improvements in access, comprehensiveness, and quality of care for individuals with mental health conditions represents a protracted and ongoing endeavor. The CDE-PHM model has certain theoretical value and practical significance for enhancing the awareness and ability of mental disorders management, helped realize the accurate management of mental health disorders in the community among the elderly in China, but also make positive attempts to cope with population aging and achieve full life cycle health management. In the subsequent promotion, the CDE-PHM model will continue to be popularized in mental health, its theoretical and practical value will be explored, and its results and academic perspectives hope to provide academic advice and a basis for other related studies.

## Availability of Data and Materials

Data not available to be shared. The raw/processed data required to reproduce the above findings cannot be shared at this time as the data also forms part of an ongoing study.
